# Functional localization within the prefrontal cortex: missing the forest for the trees?

**DOI:** 10.1016/j.tins.2010.08.001

**Published:** 2010-12

**Authors:** Charles R.E. Wilson, David Gaffan, Philip G.F. Browning, Mark G. Baxter

**Affiliations:** 1 Stem Cell and Brain Research Institute, Institut National de la Santé et de la Recherche Médicale (INSERM) Unité 846, 18 avenue du Doyen Lépine, 69675 Cedex, Lyon, France; 2 Department of Experimental Psychology, Oxford University, South Parks Road, Oxford OX1 3UD, UK; 3 Department of Neuroscience, Mount Sinai School of Medicine, One Gustave L. Levy Place, Box 1065, New York, NY 10029, USA

## Abstract

Anatomical and functional studies of the prefrontal cortex (PFC) have identified multiple PFC subregions. We argue that the PFC is involved in cognitive functions exceeding the sum of specific functions attributed to its subregions. These can be revealed either by lesions of the whole PFC, or more specifically by selective disconnection of the PFC from certain types of information (for example, visual) allowing the investigation of PFC function *in toto*. Recent studies in macaque monkeys using the latter approach lead to a second conclusion: that the PFC, as a whole, could be fundamentally specialized for representing events that are extended in time. The representation of temporally complex events might underlie PFC involvement in general intelligence, decision-making, and executive function.

## Dividing the prefrontal cortex

The prefrontal cortex (PFC), the anterior portion of the frontal lobes, is thought to be involved in a group of high-level cognitive functions variously described as executive cognitive control [1,2], behavioral inhibition [3], or general intelligence [4]. Anatomical studies of the PFC have identified multiple subregions within it. This article addresses the question of whether PFC functions should be localized to those subregions, or if the PFC has an overall function in which all regions play a part. We argue that in fact both are true. We show that subregions of PFC can be functionally dissociated. We also present evidence that there is a further overall role of the PFC in processing of temporally complex events, and this is not accounted for by adding together localized subregional functions. The initial argument derives from our own data on the effects of lesions in macaque monkeys, but these ideas are consistent with other methodological approaches to studying PFC function.

**Figure 1:**
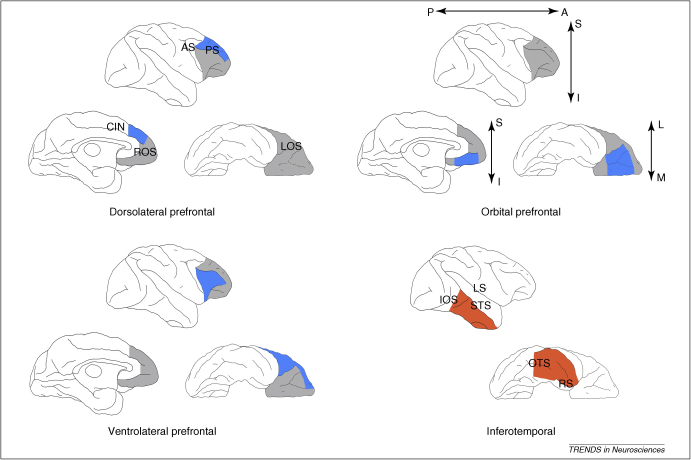
Anatomical location of the macaque monkey cortical regions discussed in this article. Top row and bottom left: the prefrontal cortex (PFC) and three subregions (dorsolateral, ventrolateral and orbital PFC) referred to in this article. The combination of grey and blue represents the whole of the PFC in each case. Blue represents the subregion in question. The subregions illustrated are those used for lesion boundaries in a number of studies discussed here [36–38], and are meant to be illustrative rather than definitive. As we point out, the PFC can be divided in multiple ways. Bottom right: the red region indicates the location of inferotemporal cortex (IT) in the macaque brain. The role of the interaction between the PFC and the IT is discussed in the latter part of this article. Directional indicators: A, anterior; I, inferior; L, lateral; M, medial; P, posterior; S, superior. Abbreviations: AS, arcuate sulcus; CIN, cingulate sulcus; IOS, inferior occipital sulcus; LOS, lateral orbital sulcus; LS, lateral sulcus; MOS, medial orbital sulcus; OTS, occipitotemporal sulcus; PS, principal sulcus; ROS, rostral sulcus; RS, rhinal sulcus; STS, superior temporal sulcus.

A number of authoritative reviews of PFC anatomy and connections exist [5–10], and therefore we do not reproduce the details here (but see Figure 1 for an illustration of the anatomical regions discussed in this article). We simply make two points about the anatomy of the PFC related to our discussion of its function. First, the PFC is clearly dissociable from surrounding cortical regions. A recent approach to PFC anatomy defines the PFC on the basis of a combination of cortical types, topology and connectivity [11]. Second, the precise localization of divisions within the PFC varies between studies; we would argue that this is because the divisions are much less clear. Indeed, one study refers to an ‘alarming disunity’ of divisional boundaries in the PFC [12], and there is notable variation in the placement and naming of cortical areas between studies, whether they are based on cell morphology and cytoarchitecture (Glossary) [8,9,13,14], or on the specific connection patterns of the regions [6,10,15–20]. Furthermore, all regions of the PFC are heavily interconnected across all divisions [21]. Thus, anatomical studies reveal a clear cortical regional unit that is the PFC, within which is contained less well-defined subregions. Here, we argue that the functional divisions mirror this pattern.

## What do the various PFC subregions do?

The localization of function in the PFC is neither straightforward nor consistent. To the extent that subregions have different connectional patterns and different physiological properties, it should in theory be possible to ascribe a discrete function to each. It is not yet clear whether this is the case. Early studies, inspired by input patterns, looked for segregation of modalities within PFC, such as separating object and spatial processing in different subregions. Such studies have had only limited success [22–25], despite suggestions to the contrary [26], and perhaps the focus is better placed on modality convergence in PFC [27].

A major source of functional evidence has been derived from neuroimaging studies on brain activation *in vivo*. Multiple neuroimaging investigations have localized specific foci of activity within the PFC that are associated with different task demands or different kinds of information processing [28,29]. A recent model draws on neuroimaging evidence to suggest that there is a hierarchical organization of function from posterior to anterior in the PFC, and that this corresponds to different levels of abstraction [29,30]. Similarly, different demands on memory processing could be associated with activation of different dorsal/ventral levels within the lateral PFC [7]. Although it is clear from neuroimaging studies that activations in distinct areas are associated with particular task demands, these data merely reflect correlations between activity and a task. Thus, even though differences in activation could be observed between subregions with high fidelity (for a recent demonstration, see Ref. [31]), this might reflect convergence of specific inputs into those subregions [32] instead of multiple, functionally-independent units.

Lesion studies are advantageous in addressing this problem because they can tell us whether a region is necessary for a particular function. Traditional lesion experiments search for double dissociations – the demonstration that a lesion to area ‘A’ impairs function one but not function two, whereas a lesion to area ‘B’ impairs function two but not one. This therefore demonstrates area-specificity and also a level of independence of these regions. Evidence of such dissociations of function within the PFC has been rare [33], with only a few studies, including two clear examples in the 1960 s, having addressed this issue [34,35]. Two recent series of lesion studies in macaque monkeys have clearly identified double dissociations of function within the PFC. The first series [36–38] demonstrated a range of dissociations and double dissociations within the PFC, for example between ventrolateral and orbital PFC in tests of strategy implementation and reward-based decision making. Another study [39] showed double dissociations between different measures of performance in a monkey version of the Wisconsin card-sorting task. This task is often used as an indicator of PFC dysfunction, and the results of this study help us to understand how and whether this task is a useful diagnostic tool.

Studies in patients with PFC damage lend support to these views, demonstrating particular patterns of impairment that coincide with damage to particular subregions of PFC [40,41]. However, patients usually have PFC damage that is not restricted to a particular subregion, and is often unilateral as well, thus it can often be hard to make definitive conclusions from lesions which have not been experimentally restricted to one specific subregion only.

## Could the PFC be more than the sum of its parts?

In contrast to studies that suggested specialization of function within the PFC, a number of influential models of PFC function emphasize its ‘adaptive coding’ properties, in which PFC neurons adapt to the demands of the task at hand, instead of carrying out a predetermined single function. Related models stress the extent to which a network of discrete regions within the PFC is recruited by diverse cognitive demands [1,4,42,43], suggesting equipotentiality of function throughout the PFC. Combined with the relative lack of double dissociations in the PFC, these observations have led to the proposal that the function of the PFC is unique and integrative [33], and that it does not have subregions with specialized functions in the way that other areas of the cortex do.

Here we summarize studies that support two opposing views: equipotentiality of function within the PFC versus a strict localization of function within the PFC, and we propose an argument that can account for both views. We accept evidence of localization, but we also argue for a global function of the PFC over and above those localized functions. This is not an argument for general equipotentiality – the differences between regions have been made clear by the recent lesion studies [36–39]. Instead, it is an argument for a specific higher order PFC function, above and beyond the localized functions. This function is not subregion-specific, and so is only revealed by studies focusing on the whole of the PFC. This idea of a system having different orders of functions is not uncommon. To take a trivial example, subregions of a car engine, such as the spark plugs, have localized functions, such as igniting fuel. But only when the engine is considered as a whole does it become clear that its higher-order function is to make the car move. However, we present evidence below that, unlike a car engine, the PFC is more than the sum of its parts – so that adding up the functions of its subregions doesn’t equate to the function of the whole. We believe this to be because the PFC is a complex and plastic system (properties discussed later on). Thus, our argument is that it is important to see the entire forest, no matter how visible the individual trees might be.

Evidence derived from lesion studies in macaque monkeys [44] provides one example to demonstrate that the PFC is doing something over and above the sum of the functions of its subregions. Monkeys with lesions of either the dorsal or ventral half of the PFC and premotor cortex were unimpaired on a test of object–reward association learning in which they learn to associate food reward with one of two stimuli [44]. By contrast, monkeys with bilateral lesions that included the entire PFC and premotor cortex were incapable of object–reward association learning [44]. The impairment is striking because this is an extremely simple learning task that both control monkeys and those with subtotal lesions find trivial [44]. It suggests that monkeys lacking the entire PFC are incapable of learning about a single object, a much more severe memory deficit than one might expect to see on the basis of the data on the role of the PFC's subregions in memory.

**Figure 2:**
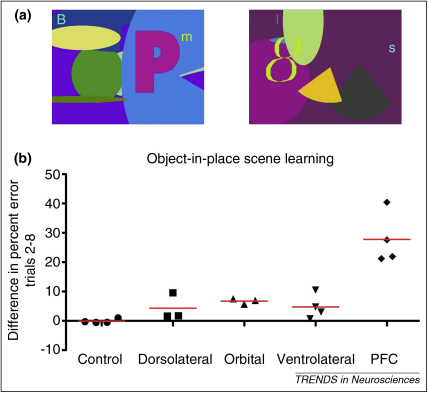
Performance of the object-in-place scene-learning task [45] following different prefrontal cortex (PFC) lesions in the macaque monkey. **(a)** Example screenshots of the object-in-place scene problems. The left and right panels show two example scenes. Each scene is composed of a colored background, a random number of colored ellipse segments, and a single large alphanumeric character (e.g., P on the left panel and 8 on the right), which collectively form the background of the scene. In this task the monkey has to learn which of the two small alphanumeric characters (objects) presented within the unique background scene is paired with a reward, and which is not (eg. B versus m in the panel on the left, and I versus s in the right panel). Objects are always in the same place in the scene, and the scene is unique to a given pair of objects. Monkeys learn about multiple problems in a given session, where one problem is a scene containing two objects. The scenes greatly aid learning, and so monkeys can acquire these problems much faster than with a plain background. Initial studies of this and similar tasks revealed that it is the combination of the unique scenes with the objects placed consistently within them that drives this fast learning [45,72]. Further, the effect is common across randomly varying scenes, and so is not dependent on any particular element of a given scene. **(b)** Comparison of performance of monkeys following subtotal and total ablations of the PFC. The data are presented as a difference score, measured as difference in percent error between pre- and post-operative performance tests. Red lines represent group means, and individual monkey scores are presented as black symbols. Monkeys with selective ablations of dorsolateral, ventrolateral, and orbital PFC all have small difference scores, but the tests do reach significance in the case of the ventrolateral and orbital ablations (see Refs [36,37] for statistical analyses). The key contrast, however, is with monkeys with complete PFC ablation, who are extremely impaired. To test our hypothesis, we performed a one-sample *t*-test on these complete PFC difference scores compared to the sum of the average deficit scores for all 3 subtotal lesions. In line with our hypothesis, this analysis revealed a significant difference [*t*(3) = 2.707, *P* = 0.037 in a one-tailed test]. Thus the complete PFC impairment is significantly greater than the sum of the impairments following the three subtotal ablations. Data taken from Refs [36–38,46].

A similar finding is evident from studies utilizing other behavioral tasks in monkeys [45] where the PFC lesions do not extend into the premotor cortex (e.g. Refs [36–39,46]). The object-in-place scene-learning task is a test of episodic memory in the monkey [45] (Figure 2a). This task is performed at chance levels by monkeys with bilateral lesions of the whole of the PFC [46]. Thus, like object–reward association learning, monkeys with bilateral lesions of the PFC are incapable of learning new object-in-place scene problems. However, even though lesions of subregions of the PFC – for example, orbital or ventrolateral – can reliably impair scene-learning [36,37], the impairments that occur after subregional lesions are a long way short of the magnitude of those that follow bilateral ablation of the entire PFC (Figure 2). Furthermore, in a test of strategy implementation, only bilateral ventrolateral PFC lesions produce a reliable impairment, but this is much smaller in magnitude than that which follows functional disconnection between the PFC and inferotemporal (IT) cortex (see below) [36–38,44]. For the purposes of the current argument, the details of these tasks are less important than the emerging pattern: across a range of tasks, functional correlates of the whole of the PFC have been demonstrated, and these are not accounted for by what we know about its subregions.

A number of studies utilizing neuroimaging techniques in human subjects are consistent with this idea. For example, a meta-analysis has shown consistent recruitment of the same network of regions in the PFC across a range of cognitive demands [43]. The authors argue that this supports specialization of function within the PFC, but of an unexpected nature, namely ‘a specific frontal-lobe network that is consistently recruited for solution of diverse cognitive problems’ [43]. The idea that large and different regions of the PFC are recruited by any task at hand supports our argument that the function of the PFC as a whole exceeds the sum of the functions of its subcomponents. The resulting question is: what is this higher-order function?

## What is the higher-order role of the PFC?

Studying the functions of the PFC as an integrated unit is difficult because of its size and diverse anatomical inputs and outputs. Bilateral lesions of the PFC are problematic in that they produce a severe generalized disruption of behavior; as we have seen, monkeys with bilateral PFC lesions are incapable of learning single discrimination problems (usually a trivial task for monkeys) over a thousand training trials [44]. Of course, this indicates the fundamental importance of the PFC in generating organized behavior, but it does not shed any light on how the PFC acts on particular kinds of information.

One way to address this problem is with selective disconnections of the PFC from particular sensory inputs using crossed unilateral lesions. This technique makes it possible to study the effects of disruption of PFC function as a whole without producing a generalized disruption of behavior [47]. For example, the inferotemporal cortex (IT, Figure 1) is a region known to represent visual object identities. In the disconnection procedure, a monkey might receive a unilateral lesion of the IT in one hemisphere and PFC in the other hemisphere. Because the connections between these structures are predominantly in the same hemisphere [27,48], this has the effect of disconnecting them from each other, while leaving the animal with functional areas of both PFC and IT. The intact IT in one hemisphere is capable of carrying out visual functions that do not depend on interaction with the PFC, and the intact PFC in the opposite hemisphere is capable of carrying out tasks that do not require the use of visual information represented in IT. The disconnection procedure (PFCxIT) therefore allows one to investigate specifically whether the PFC acts directly on inferotemporal visual information in a given task, or whether its role is less specific and is not related directly to visual representations. More generally, the crossed unilateral disconnection of PFC from other structures allows for the study of the function of the whole of the PFC within a very specific domain and without the problems associated with bilateral lesions of PFC.

Monkeys with disconnection of the whole of the frontal cortex from the IT (FLxIT) are severely impaired in a range of visual memory and strategy tasks [44,46,49–52] including delayed matching to sample, a cardinal test of recognition memory in which the monkey has to use a previously presented sample object as a cue to inform which of a subsequently presented pair of objects will be rewarded [49].

Despite the wide-ranging impairments in visual memory caused by this procedure, deficits following FLxIT cannot be interpreted in terms of a general impairment in learning about objects, as the effects of bilateral PFC lesions can [44]. This is because at least three separate studies have shown that monkeys with FLxIT or PFCxIT are not impaired at concurrent object discrimination learning [44,51,53] in which monkeys are learning to select the rewarded object from a choice pair, for several pairs concurrently. Thus, specific deficits caused by disrupting interaction between PFC and IT can be interpreted in view of the retained ability to learn associations between multiple objects and reward in a very similar task. Crossed unilateral disconnection therefore provides a more sensitive method for investigating the functional roles of the PFC.

This specific deficit that results from PFC–IT disconnection has been investigated using the discrimination learning set (DLS) task, a memory-dependent performance rule acquired during object–reward association learning, in which successive sets of discrimination problems are learned more rapidly than preceding sets. DLS is abolished by FLxIT, although the ability to learn discriminations at the speed they were learned prior to the acquisition of the learning set is not impaired [54]. This means that monkeys with FLxIT can learn the discriminations, but they are unable to improve in their level of performance in the way normal monkeys would. The improvement in task performance granted by the learning set appears to rely on prospective memory – knowledge in advance of what is coming in the next trial. So for example, if the monkey knows that object A was correct in trial *n*, and that trial *n* + 1 will contain the same choice, he can use prospective memory to choose object A on upcoming trial *n* + 1. This sort of memory requires the monkey to link information about trials separated in time, which we term a ‘temporally complex’ event.

Tasks in which problems are learned concurrently, however, do not allow the acquisition of a learning set, because successive examples of individual problems are too far apart in time to allow prospective memory formation [55]. The presence of intervening objects in the concurrent problems will block the formation of any strategy that might link instances of the presentation of a given object together. Instead, learning in this concurrent task is presumably guided by the gradual acquisition of associative strength by the objects that are being discriminated, and this memory is notably not temporally complex, requiring no information to be linked across trials.

Hence the data from these tasks are congruent with the notion that the interaction of PFC and IT is only crucial in memory during tasks requiring the processing of temporally complex events. This can be defined as an event to be learned about, in which information that is crucial to that learning is presented at more than one point in time, or that can only be interpreted with respect to a preceding event.

Each of the tasks impaired by PFCxIT cited above requires some form of temporally extended event to be remembered. The presence of unique background scenes against which each object-in-place problem is presented (Figure 2a) presumably bridges the gap between successive presentations of each individual problem, allowing rapid learning due to the fact that the scene serves as a retrieval cue for the previous encounter with each individual scene. The task is therefore temporally complex and impaired by FLxIT disconnection lesions [46]. Similarly, in delayed matching-to-sample, neither sample nor choice items can be usefully interpreted alone in relation to the task. Instead, they must be processed together as a temporally complex event for learning to occur [49]. By contrast, concurrent object discrimination learning, in which no learning set is formed, does not contain any temporally complex events and objects are gradually learned about in isolation. As such, it is not impaired by FLxIT [44].

**Figure 3:**
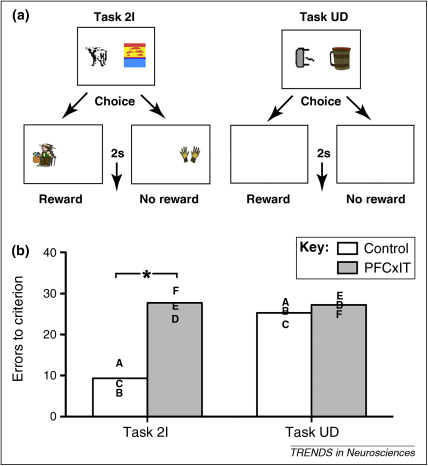
Interaction of prefrontal cortex (PFC) and inferotemporal cortex (IT) is necessary for the processing of temporally complex events. **(a)** Schematic diagram of the tasks (stimuli not presented to scale). Monkeys performed two-choice concurrent visual object discriminations in both tasks, with a 2 s delay between choice and reward delivery. In task 2I (‘two-item’), this delay was filled by another visual object on the screen, whereas in task UD (‘unfilled delay’) the delay was not associated with any visual object. See Ref. [73] for further information regarding monkeys learning about groups of stimuli. **(b)** Comparison of performance of monkeys in these visual discrimination tasks following crossed unilateral ablations of the PFC and IT (PFCxIT). Bars represent mean errors to criterion of the group, and letters represent individual monkeys’ scores that have contributed to that mean. The same letter shows the same monkey's scores in the two tasks. Monkeys with PFCxIT were impaired relative to control monkeys (with no ablations) at the 2I task, in which the choice and intervening item had formed a temporally complex event, but were not impaired at the UD task, in which all contingencies were the same as task 2I except for the fact that there was no temporally complex element. It is notable that the control monkeys find the temporally complex task (2I) easier – the sequence element presumably helping to bridge the gap to the reward, something that does not occur in the UD task. It is the loss of this facilitation that seems to cause the impairment in the monkeys with PFC/IT ablations because these monkeys perform as if there were no sequence element. Adapted from Ref. [56].

The idea that, at least in the context of processing visual information, the PFC has a general role in representing temporally complex events is a testable hypothesis, and it has received direct empirical support in two recent studies. In the first [56], specific temporal elements (in the form of an object sequence) were added to a concurrent discrimination task in macaque monkeys. Monkeys with disconnection of PFC from IT were impaired at remembering two-item sequences of visual objects, but not at the control task – concurrent discrimination with an equivalent but unfilled delay between choice and reward (Figure 3). A particularly striking feature of these data is that the impairment emerges because the control monkeys showed a facilitation at the task from the presence of the sequences relative to the unfilled delay, whereas the monkeys with disconnection did not [56]. As such, just as in DLS [54], monkeys with the PFCxIT disconnection can still learn the task, but lack the improvement in learning conferred by the ability to process temporally complex events.

The second relevant study builds upon these findings in the context of reversal learning [53]. In reversal learning, subjects learn an object–reward discrimination task, and then subsequently learn the reverse of that discrimination, in other words if A was originally rewarded, B is rewarded in the reversal phase. This behavioral task requires inhibition of the previously-learned association in order to learn the new one. Reversal learning is regarded as a cardinal function of the PFC, and there is a substantial body of evidence for a broad inhibitory role of the PFC in humans [3,57–59] and in monkeys [60–62]. This new study in monkeys directly contrasted serial learning with a learning set and concurrent learning without a learning set (as discussed in the DLS study above). The difference in this case is that the learning was occurring in the context of problems that were reversing their contingencies, rather than in the context of several different problems, as was the case for the previous examples discussed so far. Monkeys with PFCxIT were surprisingly unimpaired at concurrent reversal learning, but were impaired at serial reversal learning with a learning set [53]. This is a mirror of the DLS result above but in the context of reversal learning; again a very striking contrast between the two very similar tasks was observed. This demonstrates that, whatever the role of the PFC in visual reversal learning, it does not appear be functioning to inhibit visual representations, otherwise both tasks should have been impaired [53]. In fact, the very specific deficits in this study, and those cited above, are difficult to explain using a number of commonly cited theories of PFC function, for example inhibitory control (e.g. Ref. [3]). Thus, an additional specific explanation is required that goes beyond concepts such as cognitive control or executive function.

Support for these ideas can be derived from the properties of neurons that display activity through delay periods when no task-relevant sensory stimuli are present. Many investigators have produced general theories of PFC function based on the idea that the function of such activity is to maintain representations of sensory stimuli when they are not present [63], and this work has been linked, for example, to the concept of working memory [64]. In particular, one hypothesis describes PFC function in terms of the temporal organization of behavior [65], and suggests that the properties of PFC cells allow the PFC to ‘ensure coherence and purpose in temporally extended structures of behavior’. In addition, some approaches to subregions of the PFC, such as the idea that orbital PFC contributes to decision making by reducing the value of rewards that will not be obtained immediately (delay discounting), directly support this sort of a role for the PFC [66]. This is an example of how the current argument for a global role for the PFC can account for some established localized functions. In other words, in order to make a decision as to whether to wait for something it is necessary to be able to process temporally complex events.

In the PFC disconnection studies cited above it is important to note that in every case the disconnection has been from the whole of the prefrontal or frontal cortex; we therefore stress that this technique is informative regarding the function of the PFC as a whole. Crossed unilateral disconnections have also been used to study PFC interactions with other cortical regions [67–69]. Deficits following such lesions should help in revealing the extensive and specific role of the PFC as a whole.

## Adaptive specialization of the PFC relative to other cortical areas

The idea that the function of a region as a whole could be greater than the sum of its parts is not novel in the neurosciences. Several forms of interaction between individual memory systems lead to behaviors that cannot be accounted for by a number of independent modules acting in isolation [70]. For example, the impairment in visual learning caused by the combined ablation of the fornix, anterior temporal lobe white matter, and amygdala in monkeys notably exceeds the effects of damage to any of those structures individually [71].

One can consider, then, that the functions of subregions of the PFC are characterized by a high degree of synergistic interaction such that multiple subregions must be damaged before substantial impairment emerges. This might occur by a unique capability of the PFC, such as the processing of temporal complexity. Completion of tasks sensitive to PFC damage requires some access to that processing, but it does not matter where within the PFC that processing occurs. Loss of input information from subregional lesions might cause minor impairments, but complete lesions of the PFC could result in severe impairments because there is no longer any cortex that can represent temporally complex events. The consistent and highly specific finding of a requirement for the PFC in tasks necessitating the representation of temporally complex events supports this idea.

One alternative proposal is that perhaps many of these tasks can be solved by a number of specialized strategies, each controlled by a different subregion, and the loss of one region produces small impairments (or no impairment) in behavior because other strategies can compensate. Perhaps these strategies could relate to different types of information, such as different elements of the background scenes depicted in Figure 2. However, the results we cite in support of our proposal for a higher-order PFC function emerge from tasks using a wide variety of stimulus material and behavioral learning tasks, therefore this approach would need to posit a large number of localized strategies. More generally, the emergence of convincing evidence for a global PFC function, one that cannot be localized to any particular subregion, weakens this possibility. For example, the recent studies described above [53,56] require a very specific explanation of when the PFC is required, and when it is not, in very similar tasks. Given that the anatomical disconnections in these experiments are from the whole of the PFC, no view that regards PFC function as the sum of sublocalized strategies can explain these contrasts because the monkeys with the disconnection should lose all of those strategies for each of the tasks. In addition, there is some conceptual difficulty in arguing for such a redundant system, and there is little convincing evidence from electrophysiological recordings for separate behavioral strategies being implemented on the same task between different PFC regions.

## Conclusion

The PFC is divided into subregions, and it is clear that individual functions can be assigned to each of these subregions. These individual functions, however, do not fully account for the role of the PFC as a whole. Here we have argued that the PFC as a whole has an overarching function that is not localized to any particular subregion, and we have proposed that this role is related to its involvement in the processing of temporally complex events. The loss of this ability appears to be devastating to a wide range of cognitive tasks. We argue that a complete understanding of the role of the PFC in cognition necessitates studying not just localized functions within the PFC, but also functions of the region as a whole.

## Glossary

Ablation of cortical regions: a lesion technique involving direct access to the cortex of the brain, the removal of the pia mater, and the aspiration of the grey matter.
Concurrent object discrimination learning: a learning task in which the subject learns about a number of pairs of stimuli in a concurrent fashion – i.e. learning several problems within a session. The procedure of a given trial is the same as for singly-learned object discriminations – within each pair, one of the objects is rewarded and one is not.
Crossed unilateral disconnection: a lesion pattern in which the monkey receives a lesion to one structure in one hemisphere, and a different structure in the other hemisphere. If the two structures only communicate within the hemisphere this functionally disconnects them, but leaves the monkey with one intact portion of each region.
Cytoarchitecture: the pattern of neurons within cortical layers. The patterns can vary reliably between different cortical regions, and these variations have been used to divide the cortex into multiple areas [13].
Delayed matching to sample: a test of recognition memory. Subjects see a sample stimulus, and then after a delay have to pick the sample from a choice of two presented stimuli.
Discrimination learning set (DLS): a memory-dependent performance rule acquired during object–reward association learning, in which successive sets of discrimination problems are learned more rapidly than preceding sets.
Equipotentiality: the idea that every part of a region operates in the same manner with the same function. In the context of the PFC, equipotentiality proposes a lack of subregional specialization, and a single overall mode of action.
Localization of function: the idea that specific regions of cortex have a specific function. Within the PFC, this can be regarded as the opposing view to equipotentiality.
Object-in-place scene-learning task: a measure of episodic memory in monkeys [45].
Prospective memory: memory for information to guide future events or behavior.
Strategy implementation task: in this task, monkeys have to learn a strategy whereby some objects should be chosen persistently, and some sporadically, only after the monkey receives a reward for persistent objects. The monkey can develop an optimal strategy for gaining as many rewards as possible. The ability to maintain this strategy is tested.
Temporally complex event: an event to be learned about in which information that is crucial to that learning is presented at more than one point in time, or that can only be interpreted with respect to a preceding or future event.
Wisconsin card-sorting test: subjects learn to sort cards on the basis of a given perceptual feature of stimuli on the cards, such as the color of those stimuli. When the sorting rule changes without warning, the subjects have to adapt the rule they use for sorting – for example, now sorting according to stimulus shape instead of color. Patients with prefrontal cortical damage are thought to ‘perseverate’ by adhering to the previous rule instead of adapting their behavior to the new situation.
